# Augmenting iron accumulation in cassava by the beneficial soil bacterium *Bacillus subtilis* (GBO3)

**DOI:** 10.3389/fpls.2015.00596

**Published:** 2015-08-05

**Authors:** Mônica A. Freitas, Flavio H. V. Medeiros, Samuel P. Carvalho, Luiz R. G. Guilherme, William D. Teixeira, Huiming Zhang, Paul W. Paré

**Affiliations:** ^1^Department of Plant Pathology, Agriculture and Soil Science, Federal University of LavrasLavras, Brazil; ^2^Department of Chemistry and Biochemistry, Texas Tech UniversityLubbock, TX, USA

**Keywords:** cassava, *Bacillus subtilis*, iron induction, plant growth promotion, X-ray microanalysis

## Abstract

Cassava (*Manihot esculenta*), a major staple food in the developing world, provides a basic carbohydrate diet for over half a billion people living in the tropics. Despite the iron abundance in most soils, cassava provides insufficient iron for humans as the edible roots contain 3–12 times less iron than other traditional food crops such as wheat, maize, and rice. With the recent identification that the beneficial soil bacterium *Bacillus subtilis* (strain GB03) activates iron acquisition machinery to increase metal ion assimilation in *Arabidopsis*, the question arises as to whether this plant-growth promoting rhizobacterium also augments iron assimilation to increase endogenous iron levels in cassava. Biochemical analyses reveal that shoot-propagated cassava with GB03-inoculation exhibit elevated iron accumulation after 140 days of plant growth as determined by X-ray microanalysis and total foliar iron analysis. Growth promotion and increased photosynthetic efficiency were also observed for greenhouse-grown plants with GB03-exposure. These results demonstrate the potential of microbes to increase iron accumulation in an important agricultural crop and is consistent with idea that microbial signaling can regulate plant photosynthesis.

## Introduction

*Manihot esculenta* (cassava) is a perennial shrub in the Euphorbiaceae family native to South America and cultivated primarily by small-scale farmers for its storage roots that are eaten as a vegetable (Meenakshi et al., 2010). The tuberous roots provide the third largest source of food carbohydrates in the tropics, after rice and maize. Commercial cassava is propagated from stem cuttings as roots and do not produce buds (Food and Agriculture Organization [FAO], 2013). The semi-woody species thrives on nutrient deficient soils ranging from acidic to alkaline conditions and the presence of cyanide-rich defenses minimize damage due to insect herbivory (Blagbrough et al., 2010). Moreover as a drought-tolerant crop, it is capable of growing in marginal soils and due to its wide harvesting window serves as fall-back crop in times of famine (Montagnac et al., 2009). In terms of food calories, cassava is 25–125% more efficiency per unit area-time than other staple crops such as rice, wheat, or maize (Meenakshi et al., 2010).

While this low-tech crop is readily grown by poor subsistence farmers throughout Latin America, Africa, and Southeast Asia, low-iron abundance in cassava is associated with iron deficiency, especially for women and children on a cassava-rich diet. To combat iron deficiency in poor regions of the world associated with a cassava-rich diet, plant biofortification strategies are being sought to increase root-iron availability (Sayre et al., 2011). While most soil in which cassava is grown have sufficient iron, oxidized iron, Fe^+3^ in neutral, or alkaline soils results in low iron uptake unless iron assimilation mechanisms are activated in the plant (Curie and Briat, 2003). A network of metabolic events coordinates the mobilization of iron pools in the immediate vicinity of root epidermal cells, as well as the uptake and distribution of iron within the plant. This strategy involves three steps for iron uptake: proton exudation to enhance iron mobility, reduction of Fe^3+^ to Fe^2+^, and import of Fe^2+^.

A transgenic approach to increase endogenous iron levels has been to express an iron assimilatory protein (FEA1) from an alga (*Chlamydomonas reinhardtii*) in cassava for higher iron uptake (Leyva-Guerrero et al., 2012). While in such transgenic plants, a threefold increase in root iron is observed within 6 months of planting, such lines still face approval from a patchwork of tropical regulatory agencies as well as the dissemination of such plants to millions of local farmers that currently utilize alternative cassava cultivars (Gonzalez et al., 2009).

Low-molecular weight iron-binding molecules referred to as siderophores can also facilitate iron uptake by chelating Fe^3+^ and significantly increasing the mobility of iron in the rhizosphere. In dicots, phytosiderophore-chelated Fe^3+^ can be directly shuttled into the roots without iron reduction by specific plant transporters (Curie and Briat, 2003). In addition, soil microbes produce and release siderophores (Neilands and Leong, 1986) that are proposed to facilitate iron mobility in the soil and uptake of iron by plants (Bar-Ness et al., 1992; Glick et al., 1999). *Bacillus subtilis* (GB03) is a commercially available plant-growth promoting rhizobacterium (PGPR) strain that can be introduced into the soil at the time of planting via seed coating since spores are stable over time (Choi et al., 2014). Unlike many plant-growth promoting rhizobacterial strains that activate plant growth by directly producing and releasing indole-3-acetic acid and/or gibberellins, GB03 emits a bouquet of volatile metabolites, devoid of classic phytohormones that are capable of triggering plant growth promotion (Ryu et al., 2003; Paré et al., 2005). These volatile organic compounds have been shown to activate differential expression of approximately 600 transcripts related to cell wall modifications, primary and secondary metabolism, stress responses, hormone regulation, and iron homeostasis (Ryu et al., 2003; Farag et al., 2006; Zhang et al., 2007). *Arabidopsis* profiling of GB03-induced transcripts has resulted in a new paradigm for PGPR-mediated iron uptake. While some soil microbes are proposed to enhance iron mobility and uptake via production of bacterial siderophores (Neilands and Leong, 1986; Bar-Ness et al., 1992; Glick et al., 1999; Sharma and Johri, 2003), GB03 enhances *Arabidopsis* iron accumulation via activation of the plant’s own iron acquisition machinery (Zhang et al., 2009). Mechanistic studies reveal that GB03 transcriptionally up-regulates the Fe-deficiency-induced transcription factor 1 (FIT1) that is necessary for GB03-induction of ferric reductase FRO2 and the iron transporter IRT1 (Zhang et al., 2009). Given the important role of iron in the synthesis of enzymatic machinery for photosynthesis, the potential role of GB03 in cassava iron assimilation and photosynthesis has been investigated. Herein is reported the effect of GB03 on growth promotion, iron accumulation, and photosynthetic efficiency in soil-grown cassava plants.

## Materials and Methods

### Bacterial Cultures and Plant Treatment

*Bacillus subtilis* strain GBO3 was maintained on half-strength Murashige and Skoog (1962) solid media prepared with 1.5 % (w/v) sucrose and 0.8% (w/v) agar. For plant treatment, GB03 was streaked into MCF liquid medium (1 L) containing yeast extract (13.8 g), K_2_HPO_4_ (2.5 g), anhydrous KH_2_PO_4_ (1 g), NaCl (2.5 g), sucrose (6.5 g), manganese sulfate (0.1 g), and magnesium sulfate (0.25 g) an incubated at 25°C for 48 h. The bacterial suspension was then media diluted to an OD_570_ nm absorbance of 0.7.

Commercially grown cassava (*Manihot esculent* cv. IAC576-70) was field harvested locally and stems were cut into equal sizes (*ca*. 20 g) so as to contain only one bud per cutting. Stem sections were surface sterilized by immersion in ethanol 70% for 30 s, hipochloride 0.5% active chloride for 2 min and water rinsed (2X) before being treated with GB03 suspension or MCF. Treated one-bud cuttings were individually immersed in the bacterial suspension in a volume enough to fully cover the stem surface for half an hour then rinsed with distilled water and cuttings immersed in MCF media alone were used as a control. Treated shoots were planted after 12 h in 10-L pots containing a soil: sand (4:1) mixture. Chemical analysis of the soil substrated indicated the following elementary composition (mg dm^-3^): potassium 16, phosphorus 2.3, calcium 2.4, zinc 0.8, iron 32.1, manganese 23.7, copper 1.9, boron 0.2, sulfur 23.1, and organic matter 1.2; pH was 6.3. Plants were grown for 140 days during the summer in a greenhouse located in Lavras, MG Brazil at 21°14′ 43 south latitude and 44°59′59 west longitude with an altitude of 900 m. Temperature ranged from 20 to 30°C with relative humidy of 40–60%. Pots were arranged in a completely randomized design with three replicates per treatment. Each pot consisted of three buds with the two buds that started to produce leaves last being removed within the first week of the experiment.

### Plant Growth Measurements

Leaf physiological parameters on the fifth fully expanded leaf including photosynthesis (A) and transpiration (E) were obtained 140 days after cassava planting using an infrared gas analyzer (IRGA) ACL model PRO l (Analytical Development Co. Ltd, Hoddesdon, UK; Silveira et al., 2012). Immediately after collecting non-distructive measurements, plants were harvested for plant height, shoot dry weight and iron content via spectrophotometric and X-ray microanalysis. At the time of leaf sampling, plants were visually examined to estimate for iron deficiency (i.e., leaf chlorosis) and senescence (i.e., leaf necrosis).

### Iron Concentration Measurements

Plant iron levels were determined as described (Lobreaux and Briat, 1991). Glassware was washed thoroughly with tap water and then deionized water to avoid iron cross contamination between sample preparations. The same leaf used to measure photosynthesis was sampled for iron concentration measurements; while only making contact with the petiole, the leaf was wrapped in aluminum foil and stored in polystyrene for iron analysis. Leaves (0.5 g) were ground with a mortar and pestle in liquid N_2_, mineralized according to Beinert (1978) and reduced with thioglycolic acid. The Fe^2+^-*O*-phenanthroline complex was measured spectrophotometrically at 510 nm and iron concentration was reported on a tissue dry-weight basis using a wet-weight conversion factor determined by weighing the tissue aliquot before and after desiccation at 100°C.

Localized leaf iron content was determined by X-ray microanalysis for the fifth fully expanded leaf of each analyzed plant. From each collected leaf, two fragments of the leaf blade (3 mm × 3 mm) were excised from the middle part of the middle leaflet and processed for scanning electron microscopy. The two specimens obtained for each replicate were mounted on stubs, the leaf samples were adhered to the stubs with adhesive double sided carbon tape, one of the disks was mounted with exposed adaxial surface and the other with the abaxial surface. Samples were then covered with carbon and observed by scanning electron microscope. All mounted specimens were analyzed in Leo 040 Evo and an image of each sample surface was generated and digitally recorded, under the conditions of 20 kv and work distance of 9 mm and using Espirit 1.9 software (Bruker, Madison, WI, USA).

### Experimental Design and Statistical Analysis

All data were subjected to analysis of variance (ANOVA), using the statistical program SISVAR (Ferreira, 2011) and for significant effects, means compared by Tukey test at 5% probability.

## Results

**FIGURE 1:**
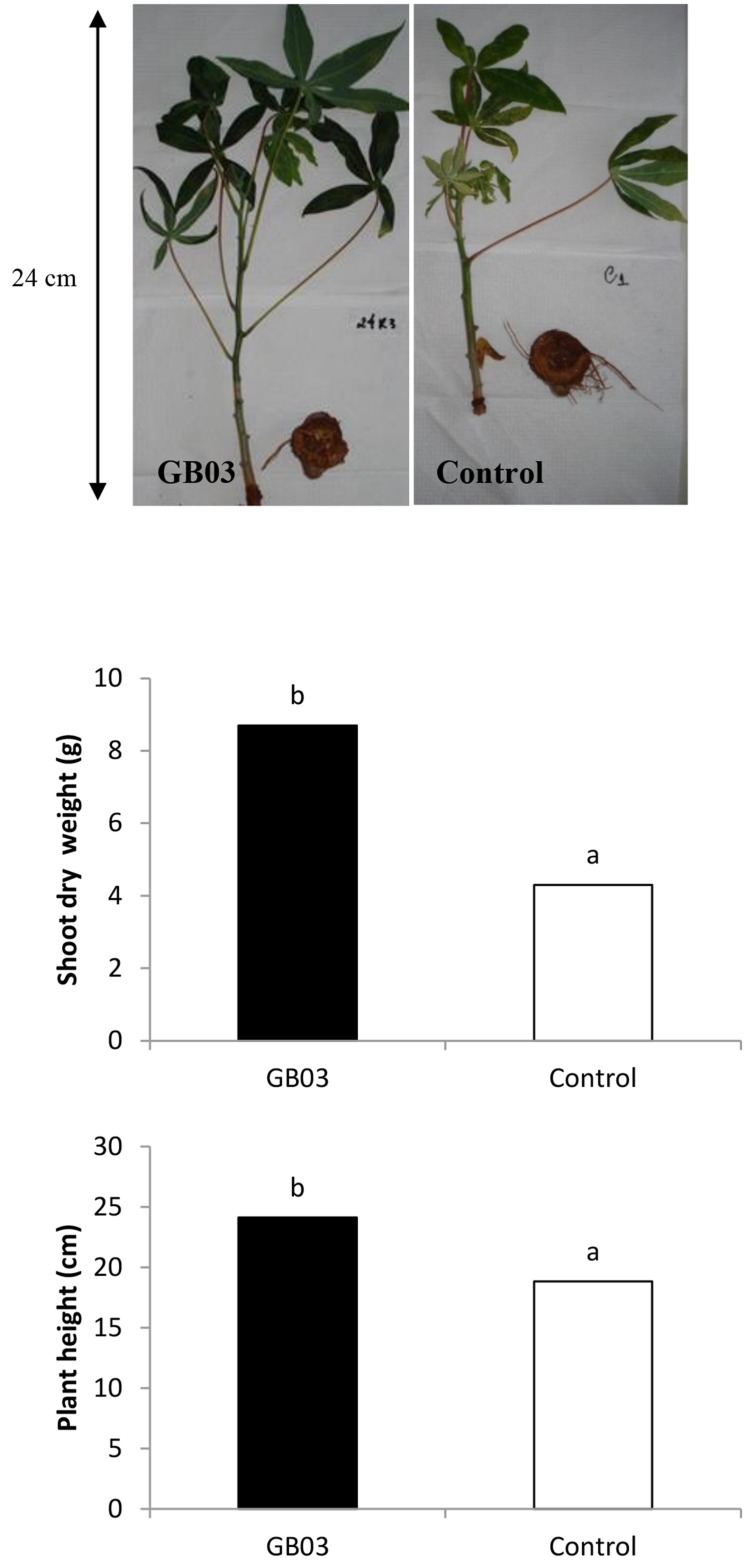
**Growth promotion of cassava (*Manihot esculenta*) cv. IAC 576-70 mediated by *Bacillus subtilis* GBO3 compared to the water control.** Mean (*n* = 3) shoot dry weight and plant height at 150 days after planting.

Since inducible iron uptake by *B. subtilis* (GB03) is associated with growth promotion in the model plant *Arabidopsis*, growth parameters including plant height and biomass were measured in cassava plants exposed to GB03. Surface-sterilized shoot cuttings, inoculated with GB03 or water (as a control) were grown in soil and harvested after 140 days. Although all plants were grown under the same environmental conditions in terms of light, soil-nutrients, and water, GB03-treated plants exhibited significant growth promotion with respect to plant height and total above-ground dry biomass at 28 and 59% greater values, respectively, when compared with GBO3 untreated plants (**Figure 1**). As a first approximation, cassava plants exhibiting increased height, branching, and shoot biomass can be correlated with greater root yields (Ntawuruhunga and Dixon, 2010) albeit harvest index (root biomass/total biomass) is the preferred parameter with breeders for cultivar selection. GBO3-treated plants also exhibited a delay in leaf senescence and abscission. Moreover, external symptoms of nutrient deficiency were less visible in GB03-exposed plants compared to water controls. Such visual indicators of micronutrient deficiency included leaf interveinal chlorosis that is symptomatic of iron deficiency and development of leaf necrotic spots/early defoliation symptomatic of senescence (**Figure 1**).

**FIGURE 2:**
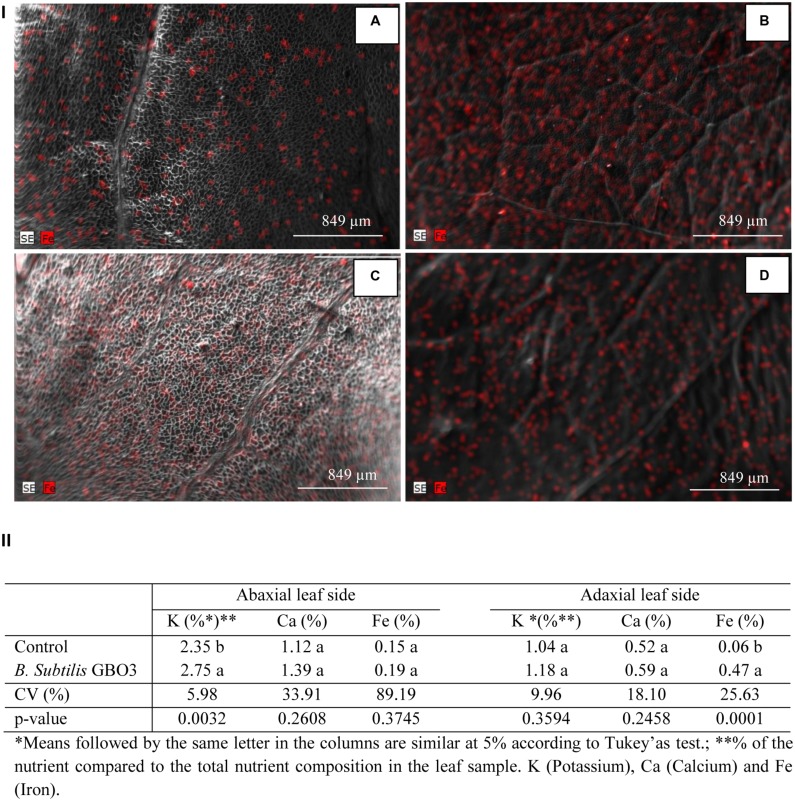
**Scanning electromicrography X-ray analysis of **(I)** the abaxial **(A,C)** and adaxial **(B,D)** leaf sides of cassava (*M. esculenta*) bacterized with *B. subtilis* GBO3 **(A,B)** or water (C,D)**. Red colored dots represent the presence and abundance of iron in the leaves; **(II)** Contents of iron (Fe), potassium (K), and calcium (Ca) in the adaxial and abaxial side of cassava (*M. esculenta*) determined by the Espirit 1.9 software from the X-ray generated images.

In addition to GB03-induced plant-growth promotion, cassava plants were chemically analyzed for increases in iron accumulation as has been previously reported with *Arabidopsis* (Zhang et al., 2009). Spectrophotometric analysis of iron in the aerial portions of the plant showed almost a 400% increase in iron abundance with GB03 treatment than water controls (*p* = 0.0004). Since photosynthetic machinery can be unequally distributed in leaves with respect to the abaxial and adaxial surface (Fernández et al., 2008), scanning electromicrograph X-ray analysis was performed to detect nutrient abundance in highly localized foliar regions. Qualitative analysis of such foliar images, regardless of the considered treatment, indicated greater iron accumulated on the adaxial versus abaxial leaf surface, in an apparently random distribution throughout the leaf surface, not in concentrated leaf regions such as the spongy cells in the vicinity of veins, as previously reported for *Cornus stolonifera* (Lambert et al., 2006). Interestingly with GBO3 exposure, leaf-side differences in iron abundance increased, with an eightfold greater adaxial iron levels and no significant iron change on the abaxial side for GB03-treated plants (**Figure 2**). Foliar microanalysis for other nutrients did not exhibit GB03 inducibility, except for potassium in which a 17% increase with GB03 treatment was observed on abaxial leaves (**Figure 2**).

**FIGURE 3:**
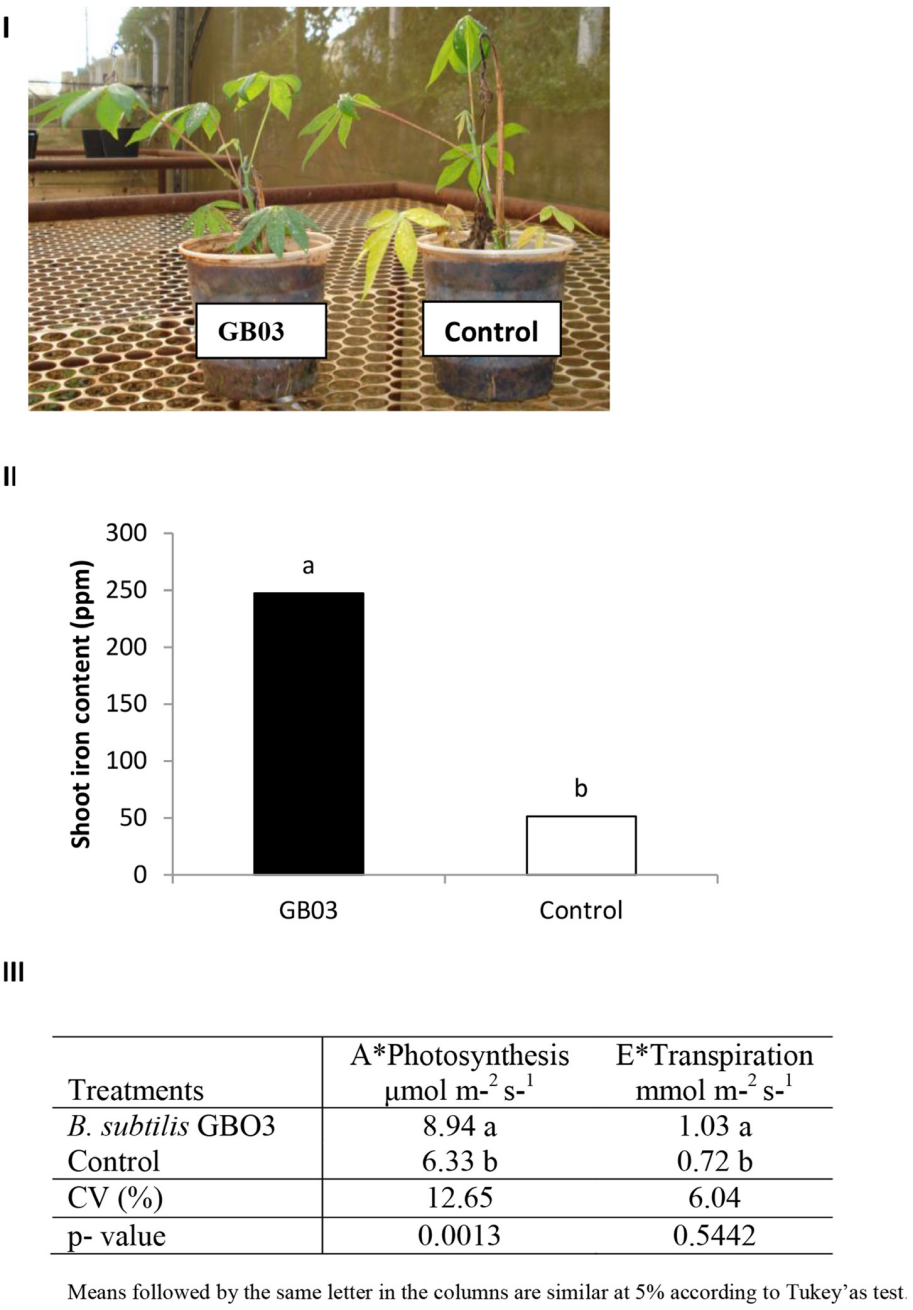
***Bacillus subtilis* GB03-mediated iron accumulation and changes in photosynthesis and transpiration compared to the water-control. (I)** Cassava plants (*M. esculenta*) used **(II)** mean (*n* = 4) chemical analysis of iron content in cassava shoot and **(III)** net photosynthesis (A) and transpiration (E) measured at the fifth true leaf, for GB03 (a) or water (b) treated cassava after 150 days of planting.

Consistent with a GB03-induced increase in iron in cassava, physiological-parameters associated with energy-acquisition including net photosynthesis and transpiration increased 40 and 43%, respectively, for GB03-treated plants (**Figure 3**).

## Discussion

Plant growth promoting rhizobacteria has long been studied to promote plant benefits from disease control to plant development and increased nutrient uptake in the absence of the pathogen (Lugtenberg and Kamilova, 2009). Cassava represents a staple food in many developing countries in the world and for its production, growers sometimes face both reduced yield due to the successive cultivation of the same crop without rotation and low use of fertilizers. In those regions, iron content in the daily diet is particularly low and supplement of the mineral is often a part of medical recommendation (Food and Agriculture Organization [FAO], 2013).

Chemical signals emitted by GB03 that trigger growth promotion in *Arabidopsis* have been reported (Ryu et al., 2003; Farag et al., 2006); GB03 has also been shown to induce biotic and abiotic tolerance, as well as increased nutrient uptake (Ryu et al., 2003; Paré et al., 2005; Zhang et al., 2007, 2009) in *Arabidopsis* even though GB03 was not commercialzed for this model plant. In contrast, cassava has not been previous reported to be responsive to GB03 induction. Augmented iron uptake has been demonstrated here to occur in cassava after exposure GB03. While regular iron content in cassava leaves ranges from 4 to 83 ppm (Montagnac et al., 2009), the GB03-treated could increase *ca*. 300% the maximum reported iron leaf content, an increase closer to the one obtained for transgenic cassava expressing the iron transporter FEA1 (Leyva-Guerrero et al., 2012).

Since GB03 has been shown to increase the abundance of transcripts involved in iron uptake and transport as well as induce rhizosphere acidification that directly mobilizes soluble iron (Zhang et al., 2009), future studies will begin to examine the mechanism of inducible iron accumulation in cassava. Similar to GB03 induction of iron in *Arabidopsis* (Zhang et al., 2007), iron accumulation in cassava was accompanied by an increase in the photosynthetic rate and biomass accumulation (**Figure 3**). The increase in iron was observed on the adaxial leaf side regardless of the treatment and may be linked with greater chlorophyll levels at this location (**Figure 2**). Elevated GB03-induced transpiration mobilizes iron into leaves once the metal ion has been absorbed by the roots and transferred to the xylem. The iron is translocated to the shoot through the transpiration flow in the form of complexes with organic acids (Curie and Briat, 2003) and accumulates in chloroplasts (Terry and Abadia, 1986) where most of the chlorophyll is concentrated (Fernández et al., 2008) allowing for optimal photosynthetic performance (Black and Osmond, 2003). Indeed, transpiration is generally negatively correlated with iron deficiency in plants (Larbi et al., 2006) and in cassava specifically positively correlated with plant growth (Silveira et al., 2012). In appears that GB03 exposure also delays leaf senescence which could be a result of higher iron and chlorophyll (Larbi et al., 2006) allowing leaves to remain green longer and/or GB03 suppresion of the hormone abscisic acid in shoots (Zhang et al., 2008), which can also delay senescence (Alonso et al., 1999).

While measurements of leaf iron levels greatly reduce the possibility of exogenous soil iron contamination of tissue samples, future studies will directly analyze for nutrient accumulation in the edible root portion. Interestingly, in certain regions of South America and Africa where anemia is more common (Food and Agriculture Organization [FAO], 2013), cassava leaves are consumed as part of the local diet (Heinma et al., 1998). Since GB03 has been shown to promote both whole plant growth and increased iron accumulation in leaves, the application of GB03 to soil grown cassava is expected to promote improved nutrition without the complication and constraints of transgenic crops. In addition, since iron is redistribution from leaves to roots, shoot iron accumulation is expected as a precursor for iron increases in roots (Terry and Abadia, 1986). Perhaps most importantly, since cassava is grown in widely varying edaphic condtions for usually more than 300 days before harvesting, these preliminary results require validation with field-grown plant for a full growing season.

## Conflict of Interest Statement

The authors declare that the research was conducted in the absence of any commercial or financial relationships that could be construed as a potential conflict of interest.
